# Evaluation of Stress Intensity and Anxiety Level in Preoperative Period of Cardiac Patients

**DOI:** 10.1155/2016/1248396

**Published:** 2016-03-02

**Authors:** Anna Rosiek, Tomasz Kornatowski, Aleksandra Rosiek-Kryszewska, Łukasz Leksowski, Krzysztof Leksowski

**Affiliations:** ^1^Faculty of Health Sciences, Department of Public Health, Nicolaus Copernicus University in Toruń and Ross-Medica, 85-830 Bydgoszcz, Poland; ^2^Faculty of Health Sciences, Department of Public Health, Nicolaus Copernicus University in Toruń, 85-830 Bydgoszcz, Poland; ^3^Faculty of Pharmacy, Department of Inorganic and Analytical Chemistry, Nicolaus Copernicus University in Toruń, 85-089 Bydgoszcz, Poland; ^4^Faculty of Health Sciences, Department of Rehabilitation, Nicolaus Copernicus University in Toruń, 85-094 Bydgoszcz, Poland

## Abstract

*Introduction*. The stress related to patient's stay in a hospital increases when it is necessary to perform a surgery. Therefore, the study of the phenomenon of stress intensity in hospitalized patients has become an important issue for public health.* Material and Method*. The study was conducted in University Hospital No. 1 in the cardiosurgery clinic. The study involved 58 patients who were admitted as planned to the hospital. The study used a standardized questionnaire measuring intensity of the stress and also deepened interviews with patients about stress and anxiety felt before the surgery.* Results*. The greater the patient's anxiety resulting from his state of health, the greater the intensity of stress in the preoperative period. This relationship is linear. The results of the study also made it possible to see intrapersonal factors (pain, illness, and suffering) and extrapersonal factors (anesthesia, surgery, and complications after surgery), which are causes of anxiety before surgery.* Conclusion*. The research showed high (negative) results of anxiety and stress associated with the disease, surgery, and complications after cardiac surgery. Active involvement in hospitalization elements, such as patient education before surgery, psychological support, and medical care organization taking into account patient's preferences, reduces the impact of stressors.

## 1. Introduction

Stress has become a major problem in our society. For medicine, it is an interdisciplinary problem. We can distinguish various factors, for example, endogenous, that is, the disease, or exogenous, for example, work place, environment, and socioeconomic status. The negative effects of stress contribute to a number of disorders and dysfunctions. Stress also triggers fear of the unknown, worries about patient's future, and future of his/her family. Usually, it manifests itself as headache, neck ache, choking sensation in the throat, trembling eyelids, and limbs. There are other symptoms such as tachycardia, palpitations, excessive sweating, and dry throat. List of ailments caused by stress is extensive. The largest groups are diseases of the heart and circulatory system [1].

For those reasons, the phenomenon of stress and its negative impact on the functioning of human society are interesting for many different areas including public health sector. Human functioning in isolation from stress is not possible to achieve. Therefore, in the case of the health sector to minimize its negative impact has become necessary in order to improve the health status of hospitalized patients. The stress of the patient's stay in the hospital increases when it is necessary to perform a surgery. Literature review shows that waiting for cardiac surgery evokes greater stress response than other types of surgery [2, 3]. This phenomenon is disadvantageous, since the additional stress factors may unexpectedly degrade the condition of the patient. It has been proven that the mere appearance of own illness is a difficult situation and a source of stress for the patient [4, 5]. The course of the disease in its further progress depends largely on the strength of the stress response. Severe anxiety negatively influences the physiological parameters and disrupts the postoperative period, increasing the number of complications and lengthening the time of hospitalization. We can counteract the stress reaction, by multiple antistress behaviors during the patient's stay in hospital. Family support, available psychologist on the ward patient education, healthcare organization that focused on individual patient preferences are undoubtedly challenges for the health system and public health in Poland. These actions will reflect on the quality of life of the patient and the patient's physical condition at the time of illness, but they will also be useful in the process of minimizing the severity of coexisting diseases and will help us to avoid the development of other diseases [6].

## 2. Material and Methods

The study was conducted on 58 patients who were about to undergo cardiac surgery. The results presented in the work are divided into three parts, taking into account demographic group, the overall level of anxiety and stress before surgery, and self-assessment of psychoemotional state. This vision lets us show a dependence related to the analyzed levels of stress and anxiety. The study used a standardized questionnaire about the intensity of stress related to the patient's situation (PSS-10) and deepened interviews with the patients. The test gave answers to questions about the level of feeling of anxiety in eight categories (illness, admission to hospital, anesthesia, surgery, uncertainty, pain, complications, and suffering) assessed on a four-point scale of the perceived level of anxiety (0: no anxiety, 1: slight anxiety, 2: moderate anxiety; 3: serious anxiety).

The study used two variables that were analyzed.

*Feeling of Anxiety*. The degree of anxiety experienced by the patient (psychoemotional state) based on the organizational factors in a hospital and on the disease and the demographics.

*Level of Anxiety*. Intensity of stress identified before surgery depending on occurring comorbidities, need for surgery (pain, complications, disease, and anesthesia).

We show a comparative analysis of the results obtained from the questionnaire PSS-10, in relation to the answers given by respondents regarding their psychoemotional state (feeling of anxiety). Analyzed issues included feeling anxious and experiencing the anxiety associated with health. The patients were asked which element in the organization of hospital care can help to minimize anxiety before surgery: patient education conducted on the ward (on the functioning after surgery), information on patient's health and results of the examination, talking with a psychologist before surgery, talking with patient's loved ones (family members), talking to a nurse the patient trusts, and talking with patient's doctor.

The results were statistically analyzed using descriptive statistics and using tests of statistical significance. Comparison of variables lets us specify whether a change in one trait changes another feature and whether these changes are proportional or inversely proportional to each other. The study used the Spearman correlation coefficient *R* which is shown in Table 1.

For the verification of all analyses, a significance factor at the level of *α* = 0.05 was used which allowed the variables to be considered statistically significant at level *p* < 0.05.

The Institutional BioMedical Ethics Committee approved this study, file number KB 263/2015. Written informed consent for participation in the study was obtained from participants of the study and for publication of this report.

## 3. Results

The results are discussed in correlation with the categories.

### 3.1. Demographic Data

The study group included 58 people of which 59% were men (34 subjects) and 41% were women (24 subjects).

### 3.2. Gender

**Figure 1:**
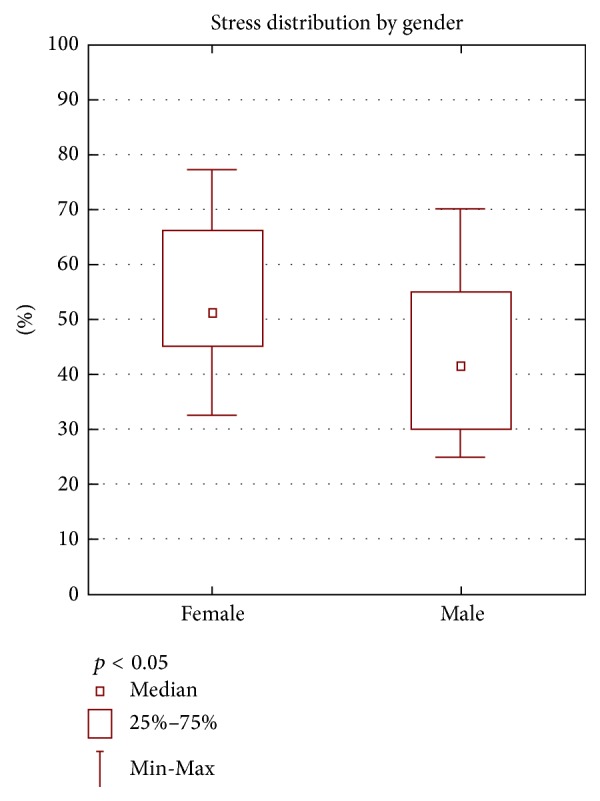
Stress distribution by gender.

Statistical analysis of the results of the questionnaire PSS-10 showed that gender in a statistically significant way affects the intensity of the stress related to the surgery and illness. Women have higher values (*p* < 0.05). The values obtained indicate that women are more likely than men to experience stress. Stress distribution by gender is shown in (Figure 1).

### 3.3. Age

The largest group was patients between 66 and 80 years (52%). The second group was patients between 52 and 65 years (39%). The smallest group was people aged between 37 and 51 (7%).

The analysis showed that the age of the respondents does not affect the feeling of stress and anxiety before cardiac surgery (*p* > 0.05). It was also found that there is no correlation between the measured parameters and age (Table 3).

### 3.4. Place of Living

Most people in the researched group lived in cities (43% of respondents). Towns were indicated by 33%, while small towns were indicated by 24%. The place of living of the respondents does not affect the feeling of stress and anxiety before cardiac surgery (*p* > 0.05).

### 3.5. Education Level

**Figure 2:**
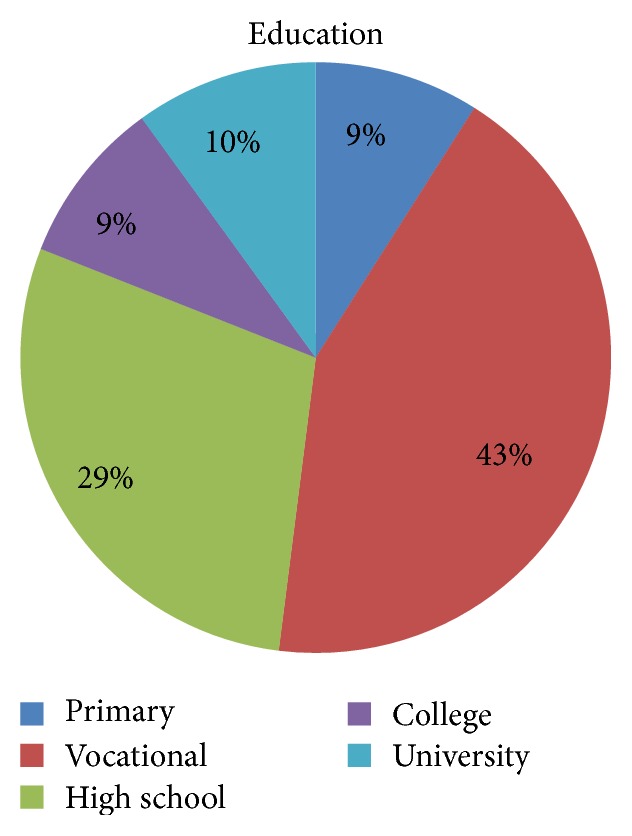
Distribution of educational groups based on levels of education in Poland.

Education level of the respondents is not uniform. There were people with primary education (9%), with university level education (10%). However, the largest group of respondents had vocational, job-related education (43%). Educational level of the respondents does not affect the feeling of stress and anxiety before cardiac surgery (*p* > 0.05) (Figure 2).

The common phenomenon in the researched patients was the presence of comorbidities (other than cardiac disease requiring surgery). They were present in 57% of respondents. Hypertension occurring in 21 patients and metabolic diseases occurring in 11 patients were among the most common comorbidities. Other illnesses were less common. Details of comorbidities in the studied group are shown in Table 4.

**Figure 3:**
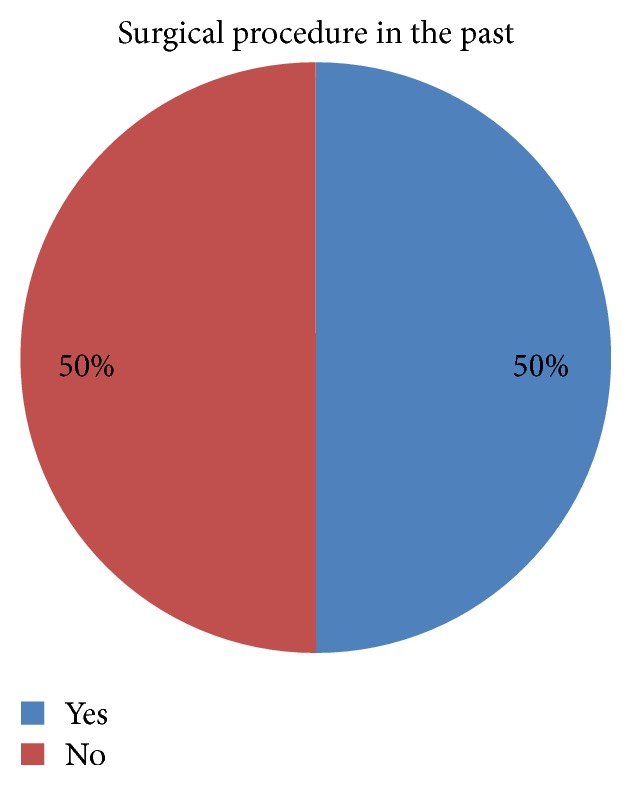
Surgical procedures in the past.

Half of the researched patients declared they underwent a surgical procedure in the past (Figure 3). However, the fact that the patients underwent a surgical procedure in the past does not affect the feeling of stress and anxiety before cardiac surgery (*p* > 0.05).

### 3.6. Analysis of the Level of Anxiety and Stress for Patients

The question about the level of feeling of anxiety concerned eight categories evaluated on a four-point scale. The obtained average results indicate that the researched patients had the greatest anxiety feel about the surgery. This is followed by anxiety the disease itself and about the postoperative complications in the third place (Table 5).

Patients are afraid of cardiac surgery, illness, and complications and are also anxious and fearful of the possible pain, suffering, and anesthesia they receive. Similar average values get questions about anesthesia, suffering, and admission to hospital. The values obtained indicate that the intensity of stress identified by patients before surgery depends on the fear of cardiac surgery, pain, complications after surgery, and anesthesia (preoperative hospital variable). Analysis of the correlation of preoperative hospital variables and patient's feeling of anxiety and stress is presented in Table 6.

The average results indicate relatively high feeling of anxiety (48% in decilc scale) and average correlation of preoperative hospital variables in the test group. The level of anxiety interpreted in decilc scale (scale from 0% to 100% every ten percent) indicates the average anxiety level of 55% and strong correlation of preoperative hospital variables.

The results of the questionnaire PSS-10 indicate the occurrence of stress on the average level of 48% with dehiscence quartile ranging from 38% to 58%. In each of the three presented variables, we can see the above-average values and a similar spread of values.

**Figure 4:**
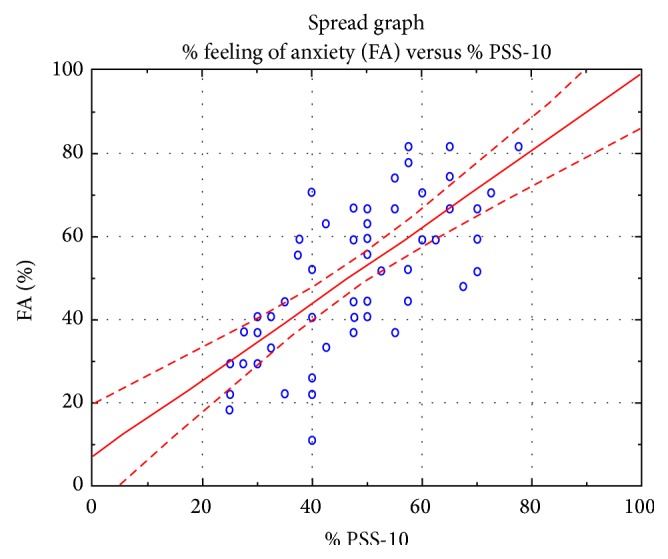
The relationships between responses about the feeling of anxiety compared to the results of the questionnaire PSS-10.

**Figure 5:**
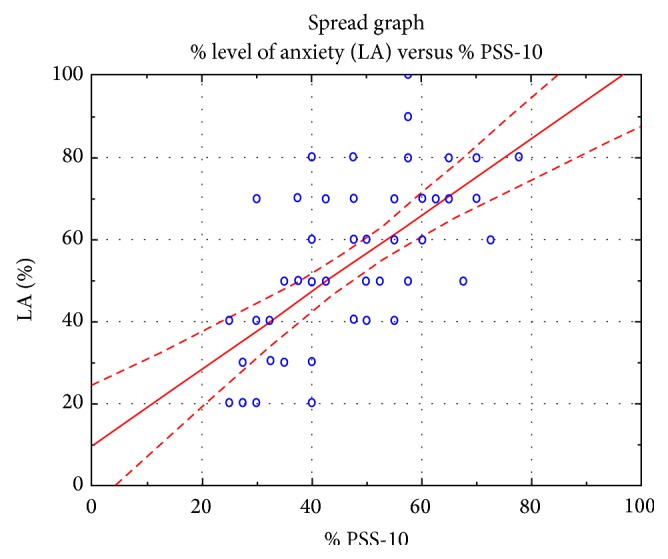
The relationship between responses about the level of the anxiety in relation to the results of the questionnaire PSS-10.

**Figure 6:**
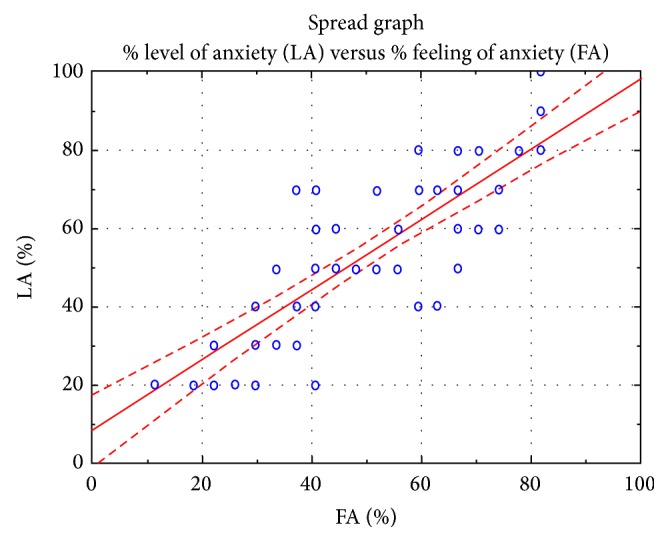
The relationships between responses about the level of the anxiety in relation to the feeling of anxiety.

The results of the study indicate a strong linear relationship between the analyzed variables, which confirms the interdependence between the analyzed features such as a feeling of level of anxiety and the results of the questionnaire PSS-10 (Figures 4, 5, and 6).

The resulting statistical analysis confirms the relationship between the questions asked during interviews and the results obtained from the questionnaire PSS-10.

In addition, in the whole group, we can observe that prevailing negative (high) results of anxiety and stress are related to illness and surgery.

### 3.7. Self-Assessment of the Patient's Psychoemotional State

#### 3.7.1. Feeling Anxious

The study showed that anxiety is felt by 28% of patients, and lack of concern is found only in 7% of the whole group.

Correlation of responses indicates a strong linear relationship that is statistically significant, which shows that the higher the values of feelings of anxiety, anxiety levels, and the values of the questionnaire PSS-10, the higher the rank from the question.

Among the analyzed variables, there is a relationship shown in Figure 8.

#### 3.7.2. Experiencing the Anxiety Associated with Health Status

The answers given by the respondents indicate that anxiety is felt by as many as 42% of patients, while only 4% of the entire group does not feel any anxiety. Intermediate values occur in 54% of the respondents.

Correlation of responses indicates a strong linear relationship that is statistically significant, which shows that the higher the values of feelings of anxiety associated with patient's medical condition, the higher the level of anxiety, the sense of anxiety, and the values from the survey PSS-10.

During the depended interviews, the patients were asked about the elements of the organization of hospital care that in patient's opinion could significantly minimize anxiety before surgery. The data are presented in Table 9.

## 4. Discussion

The research conducted on patients scheduled for cardiac surgery (Tables 2 and 3, Figures 1, 2, and 3) demonstrated the existence of stress and anxiety associated with illness and surgery. Stress associated with the current state of health occurs in 48% of the researched patients (Tables 7 and 8) (Figures 4, 5, and 6). In several studies stress associated with preoperative waiting was cited as a trigger for anxiety [7–9].

Statistical analysis of the collected data showed that only gender has a statistically significant effect on the intensity of the stress felt because of a surgery and illness (*p* < 0.05) (Table 2, Figure 1). The obtained values indicate that women results are significantly higher, which confirms the hypothesis about the impact of gender on the analyzed variables. Women more than men, suffer from stress and anxiety occurring before heart surgery. Other demographic data such as age, place of living, and education level (Table 3, Figures 1 and 2) have no a statistically significant effect on the intensity of the stress. Similar results are confirmed by other researchers. Jawaid et al. found that there is a statistically significant higher level of preoperative anxiety in females when compared to males [10].

**Figure 7:**
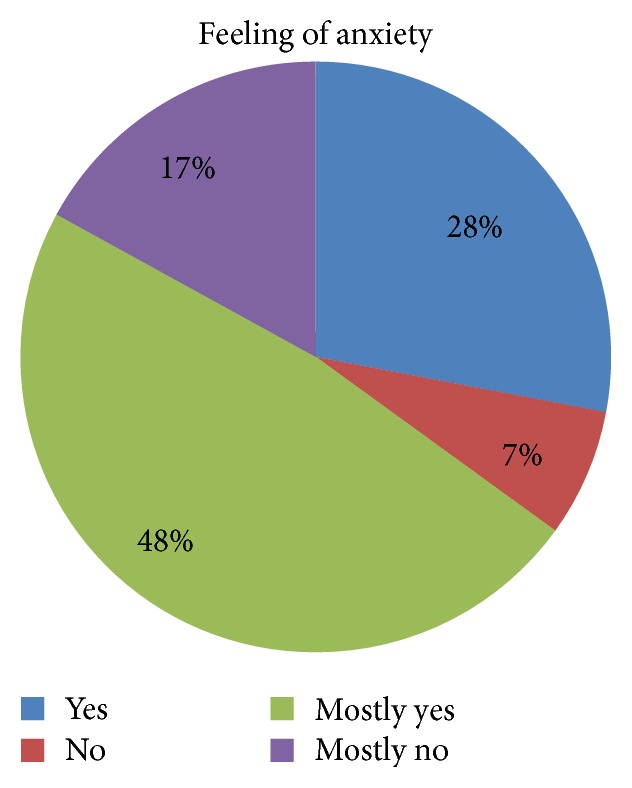
Graphical representation of the feeling of anxiety expressed by respondents.

**Figure 8:**
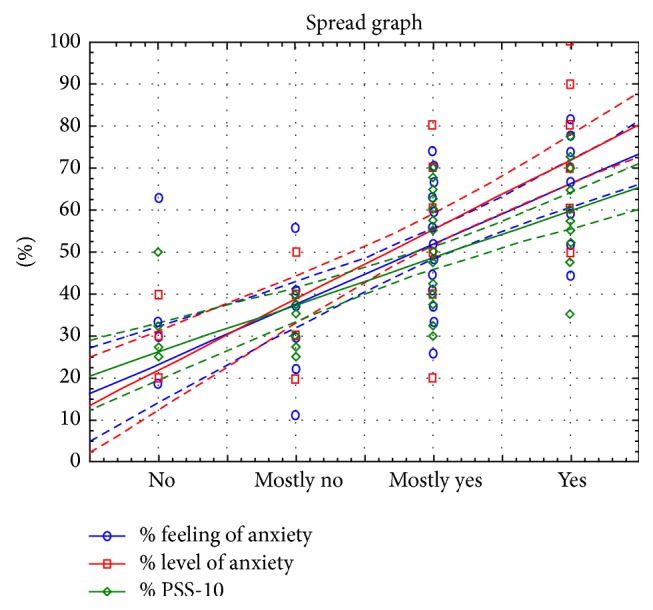
Graphical representation of the relationship between the level of feeling of anxiety and the results of feeling of anxiety, anxiety level, and the PSS-10.

Although the experience of other authors confirms that surgery causes stress and contributes to increased anxiety and a simultaneous decline in the mood of the patient [2, 3, 11], it was not confirmed in this study. Depression in the group of respondents was not diagnosed in a coexisting illness (Table 4). Patients' self-assessed psychoemotional state indicated anxiety before surgery in 28% of the respondents (Figure 7). Our study only indicates a strong linear relationship between patient's psychoemotional state and feeling of anxiety (Table 9, Figure 8).

The results of this study allowed us to showcase not only the intrapersonal factors (pain, illness, and suffering) but also the extrapersonal ones (anesthesia, surgery, and complications after surgery) which are the causes of anxiety before surgery. In the whole group, we could observe the prevailing high (negative) results for the anxiety and stress associated with the disease, surgery, and complications after cardiac surgery (Tables 5 and 6). This tendency is also present in other studies [12]. Borsook et al. also indicated that acute stress in the perioperative period has four major contributors such as anxiety, pain, surgical stress response, and anesthesia procedures [13]. Our study also indicated that, apart from these factors, also such facts as admission to a hospital before surgery provide feeling of anxiety (*p* < 0.05).

**Figure 9:**
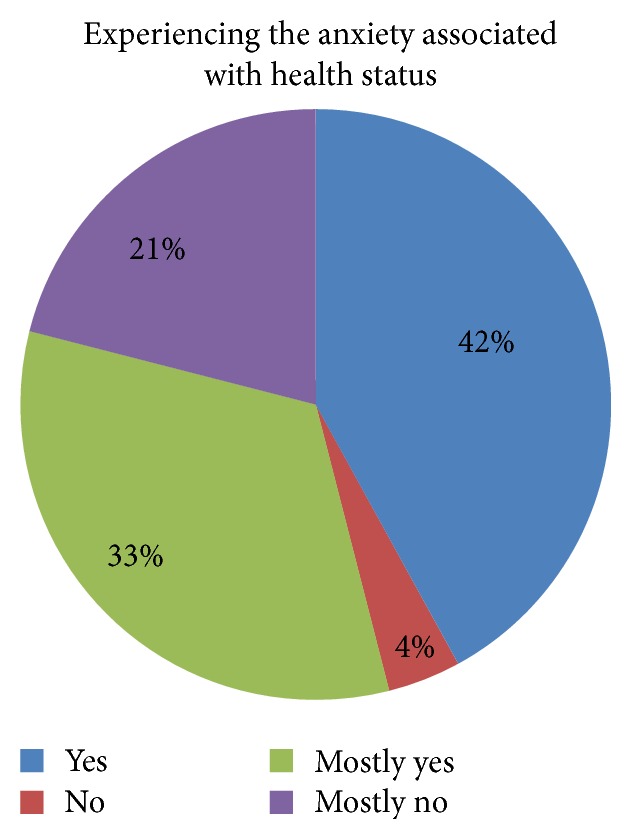
Graphical representation of the feeling of anxiety associated with health status.

**Figure 10:**
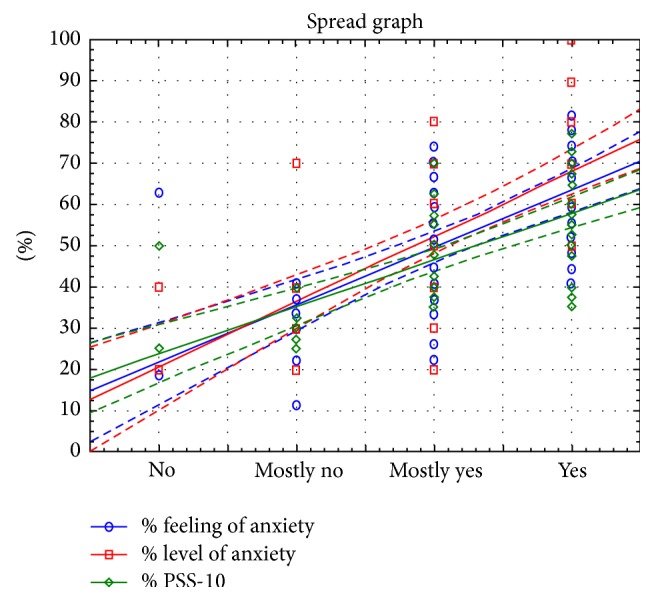
Graphical representation of the relationship between the level of feeling of anxiety associated with the state of health and the feeling of anxiety, anxiety level, and PSS-10.

Surgical procedures in the past have no influences on patients' feeling of anxiety (*p* > 0.05) (Figure 3). Surgery is a situation difficult for the patient, because she/he has no direct influence on the course of surgery and it is out of his/her control. Surgery conjures the greatest sense of fear among the respondents (average value 2.5; *p* < 0.05; Table 5). Patients are afraid of after-surgery illness (intrahospital infections) (average value 2.0; *p* < 0.05; Table 5), complications (average value 1.8; *p* < 0.05; Table 5), and are also anxious and fearful about possible pain (average value 1.6; *p* < 0.05; Table 5), suffering, and the anesthesia they receive (average value 1.4; *p* < 0.05; Table 5) and also the success of the surgery procedure (average value 1.3; *p* < 0.05; Table 5). Anxiety in surgical patients can increase the need for anesthesia, which increases anesthetic risk [14]. Patients afraid of pain, and, in consequence anxious, may require an increased dosage of postoperative pain medication, which can affect postoperative recovery because of decreased physical activity, increased risk of bowel upset or slowed respiration [11, 14]. Areas related to anxiety indicated by the patients during their stay in hospital also testify to the facts such as the following: medical staff has insufficient contact with the patient (*p* < 0.05), lack of education (*p* < 0.05), and inadequate psychological support of the patient (*p* < 0.05) (Table 11). Some patients reported insufficient contact with the medical staff and feeling that nurses were not open to their concerns. These patients felt that they were not treated as individuals while they were waiting for surgery. The same tendency is shown by other researchers. Jangland et al. [15] found that, among those patients who complained about care and increased anxiety, the most common complaints were insufficient information, inadequate respect, and insufficient empathy. These factors increased patients' anxiety and reduced their confidence in the healthcare system. The presence of these factors shows gaps in Polish healthcare system and in the organization of cardiac hospital care. Despite advanced technology implemented in the treatment of cardiac patients the preoperative elimination of stress and anxiety associated with the illness is not adequately satisfied. The study showed that 42% of cardiac patients treated surgically experience anxiety (Figure 9), which reflects the lack of appropriate patient education, lack of psychological support, and lack of information on the operation conducted on a hospital ward. Our study also confirms strong linear relationship, which shows that the higher the values of feelings of anxiety associated with patient's medical condition, the higher the level of anxiety and the sense of anxiety (Table 10, Figures 9 and 10). This tendency is also present in other studies [16, 17]. Research conducted by Gois et al. confirmed that education and communication of information to the patient and the organization of care taking into account patient's preferences minimize stress before cardiac surgery. Also previous studies [12, 18] have shown that the lack of social and psychological support for a patient is a cause of stress before surgery and is related to the risk of death within 6 months after cardiac surgery. It should be noted that the results of our research revealed that a conversation with a close person or relative before surgery is not a factor in eliminating stress before a surgery (*p* > 0.05, Table 11). Although numerous studies [19–21] show this element as essential to the elimination of stress in various life situations, in case of an operation it does not help to reduce stress. This condition of the patients is, however, fully justified because the risk of death from ischemic heart disease in patients with anxiety doubles. In order to eliminate the risk of premature death in patients treated surgically, we should eliminate stress and lower the anxiety levels during hospitalization. For this purpose, it is necessary to extend the hospital's care for cardiac patients. This area requires the inclusion of patient education process (*p* < 0.05) and the inclusion of psychological care (*p* < 0.05), as well as the organization of healthcare which would take into account patient's preferences. Patient education in preoperative period and reliable information given to the patient reduce stress and help him/her to master anxiety. Spaulding in his research found that preoperative education reduces anxiety because it gives the patient a sense of what to expect [11]. The legitimacy and importance of the educational process are also shown by other researchers [12, 22, 23].

Factors related to the organization of hospital care have however a direct impact on eliminating the stress of a patient before surgery.

## 5. Conclusion

Stress and anxiety were present in cardiac patients in the preoperative period. The results of this study allowed us to showcase not only the intrapersonal factors (pain, illness, and suffering) but also the extrapersonal ones (anesthesia, surgery, and complications after surgery) which are the causes of anxiety before surgery. The high intensity of stress and anxiety level in the preoperative period of cardiac patients depend on the gender of the patient. Women are more often afraid of cardiac surgery than men. The study showed that the greater the patient's anxiety resulting from his state of health, the greater the intensity of stress in the preoperative period. This relationship is linear. Conducted research revealed areas of healthcare organization that need improvement in order to reduce stress in cardiac patients on the ward. These include education of the patient before surgery, psychological support, and organization of care that takes into account patient's preferences. Active inclusion of these elements in the process of hospitalization can reduce the impact of stressors and their negative impact on homeostasis. However, further research is needed about mechanisms by which anxiety, education, or other psychosocial factors may be related to objective health endpoints of cardiology patients.

## Figures and Tables

**Table 1:** Determination of the coefficient of correlation in relation to the strength of the association.

Correlation coefficient *R*	The strength of the correlation relationship
0.0–0.3	Lack
0.3–0.4	Weak
0.4–0.7	Average
0.7–0.9	Strong
0.9–1.0	Very strong

**Table 2:** Gender and level *p*.

Gender		SD	Min	*Q*1	Me	*Q*3	Max	Level *p*
Female	55%	13%	33%	45%	51%	66%	75%	0.003
Male	44%	13%	25%	30%	41%	55%	70%	

**Table 3:** Correlation between measured parameters (feeling of anxiety and level of anxiety) and age.

Age	Correlation of the Spearman rank order			
	Important	*R* Spearman	*t*(*N* − 2)	Level *p*
Feeling of anxiety	58	−0.183	−1.379	0.174
Level of anxiety	58	−0.098	−0.734	0.466
PSS-10	58	−0.030	−0.226	0.822

**Table 4:** Types of coexisting diseases in researched patients.

Coexisting diseases	Number of patients
Metabolic diseases (diabetes type II)	5
Diabetes type II and hypertension	6
Hypertension	6
Hypertension, sclerosis, and hyperlipidemia	5
Hypertension and cardiac agrest	4
POChP	4
Cardiac agrest	4
Cataract	1
Cancer	1
Parkinson's diseases	1

**Table 5:** Perioperative factors influencing the feeling of anxiety and stress in eight categories.

Variable	Medium value	Level *p*
Surgery	2.5	*p* < 0.05
Illness	2.0	*p* < 0.05
Complications	1.8	*p* < 0.05
Pain	1.6	*p* < 0.05
Suffering	1.4	*p* < 0.05
Anesthesia	1.4	*p* < 0.05
Admission to hospital	1.4	*p* < 0.05
Incertitude	1.3	*p* < 0.05

**Table 6:** Analysis of the correlation of preoperative hospital variables and patient's feeling of anxiety and stress.

Perioperative hospital variables	Correlation of the *R* Spearman rank order	
	Feeling of anxiety	Level of anxiety
Surgery	0.87	0.93
Illness	0.75	0.70
Complication	0.65	0.68
Pain	0.70	0.65
Suffering	0.63	0.61
Anesthesia	0.80	0.61
Admission to hospital	0.60	0.58
Incertitude	0.59	0.60

**Table 7:** The results of descriptive statistics of the questionnaire PSS-10, the level of anxiety on a decilic scale, and total anxiety associated with the current state of health.

Variables	*N*	$\overset{-}{x}$	SD	Min	*Q*1	Me	*Q*3	Max
% PSS-10	58	48%	14%	25%	38%	49%	58%	78%
% level of anxiety	58	55%	20%	20%	40%	55%	70%	100%
% feeling of anxiety	58	51%	18%	11%	37%	52%	67%	81%

**Table 8:** Correlation table of the results of the level of anxiety, the feeling of anxiety, and the values from the questionnaire PSS-10.

Variables	Correlation of the Spearman rank order	
	% feeling of anxiety	% level of anxiety
% level of anxiety	0.81	N/A
% PSS-10	0.71	0.65

**Table 9:** Analysis of the correlation of variables in relation to feeling anxious.

Feeling anxious	Correlation of the Spearman rank order			
	Important	*R* Spearman	*t*(*N* − 2)	Value *p*
% feeling of anxiety	58	0.70	7.33	0.001
% the level of anxiety	58	0.70	7.26	0.001
% PSS-10	58	0.70	7.35	0.001

**Table 10:** Analysis of the correlation of variables in relation to the feeling of anxiety associated with patient's medical condition.

Feeling of anxiety associated with patient's medical condition	Correlation of the Spearman rank order			
	Important	*R* Spearman	*t*(*N* − 2)	Value *p*
% feeling of anxiety	58	0.67	6.76	0.001
% the level of anxiety	58	0.67	6.76	0.001
% PSS-10	58	0.71	7.53	0.001

**Table 11:** Essential elements of a healthcare organization that could minimize patient's stress before surgery.

Variable	Level *p*
Cardiac patient education conducted on the ward (e.g., on the functioning after surgery)	*p* < 0.05
Information on diagnosis provided by medical personnel	*p* < 0.05
Interview with a psychologist before surgery	*p* < 0.05
Talk with a nurse (before treatment) in whom the patient has confidence	*p* < 0.05
Talk with patient's doctor	*p* < 0.05
Conversation with a loved one (family member)	*p* > 0.05
